# Temporary loss of consciousness during cetuximab treatment of a patient with metastatic colon cancer: a case report

**DOI:** 10.1186/s40792-019-0707-5

**Published:** 2019-10-21

**Authors:** Taro Fukui, Koichi Suzuki, Sawako Tamaki, Iku Abe, Yuhei Endo, Hideki Ishikawa, Nao Kakizawa, Fumiaki Watanabe, Masaaki Saito, Shingo Tsujinaka, Kazushige Futsuhara, Yasuyuki Miyakura, Hiroshi Noda, Toshiki Rikiyama

**Affiliations:** Department of Surgery, Saitama Medical Center, Jichi Medical University, 1-847 Amanuma-cho, Omiya-ku, Saitama, 330-8503 Japan

**Keywords:** Temporary loss of consciousness, Cetuximab, Serum magnesium, Colon cancer

## Abstract

**Background:**

Anti-epidermal growth factor receptor (EGFR) antibody is widely used for the treatment of patients with metastatic colorectal cancer. Hypomagnesemia is a comparatively frequent adverse event of this drug, which is likely overlooked because it occurs later in treatment without symptoms. Furthermore, hypomagnesemia and hypomagnesemia-induced corrected QT (QTc) prolongation may lead to loss of consciousness (LOC), the onset of which is not generally considered associated with the treatment of anti-EGFR antibody because of its rare occurrence. Here, we present a colorectal cancer patient treated with anti-EGFR antibody, who suffered LOC during treatment while severe hypomagnesemia or QTc prolongation was not observed.

**Case presentation:**

A 69-year-old man with metastatic colon cancer was treated with cetuximab (anti-EGFR antibody) plus irinotecan as third-line chemotherapy. His serum magnesium level gradually decreased, and grade 2 hypomagnesemia (a serum magnesium level of 0.9 mg/dL) was observed at the 12th administration of cetuximab. In light of this development, intravenous supplementation of 20 mEq magnesium sulfate began with careful blood monitoring despite the lack of clinical symptoms. Electrocardiogram (ECG) showed prolonged QT or corrected QT (QTc) intervals (grade 1). His serum magnesium level remained at 0.9 mg/dL, and no hypomagnesemia symptoms were observed by the 17th administration of cetuximab. After the treatment, however, he suddenly lost consciousness without symptoms related to infusion or allergic reactions. Circulatory collapse following dermatological reactions and respiratory events were not evident. Intravenous supplementation of magnesium sulfate was administered again. He awakened 2 min after the onset of temporary LOC without any other symptoms related to hypomagnesemia, such as lethargy, tremor, tetany, and seizures. No other etiology outside of the low level of serum magnesium was confirmed in further examinations. Cetuximab was discontinued, and his serum magnesium level returned to a level within the normal range after 6 weeks. Because of tumor progression, regorafenib and TAS-102 (trifluridine tipiracil hydrochloride) were introduced sequentially for 6 months. Five months after the final treatment of TAS-102, he died of his primary disease, which reflected a survival period of 4 years and 6 months since the beginning of treatment.

**Conclusions:**

This case report reminds clinicians that LOC can be induced without severe hypomagnesemia or QTc prolongation, during anti-EGFR antibody treatment for metastatic colorectal cancer even while under carefully monitored magnesium supplementation.

## Background

Combination therapy comprising anti-epidermal growth factor receptor (EGFR) antibody and anti-cytotoxic drugs has shown a survival benefit as a first-line [1], as well as a second-, third-, and salvage-line chemotherapy [2–7] for patients with metastatic colorectal cancer (mCRC) without RAS mutations. The adverse event of hypomagnesemia is comparatively frequent with anti-EGFR antibody treatment [8] but is likely to be overlooked because it occurs in later treatment periods, and hypomagnesemia is asymptomatic until it becomes severe [9]. Furthermore, hypomagnesemia and hypomagnesemia-induced QTc prolongation may lead to a loss of consciousness (LOC) [10–13], but few papers have reported the association of anti-EGFR antibody treatment with the induction of loss of consciousness (LOC) [14, 15]. Herein, we present a mCRC patient treated with anti-EGFR antibody, who showed LOC during treatment while severe hypomagnesemia or QTc prolongation was not observed.

## Case presentation

A 69-year-old man with ascending colon cancer and multiple liver metastases was treated with cetuximab plus irinotecan as third-line chemotherapy. Because of bowel obstruction symptoms such as abdominal pain and vomiting, he had undergone laparoscopically assisted right hemicolectomy with D3 lymph node dissection followed by chemotherapy. The pathological finding revealed well-differentiated adenocarcinoma. Sequential treatments were carried out, including XELOX (capecitabine 2000 mg/m^2^/day p.o., twice daily for 14 consecutive days; oxaliplatin 130 mg/m^2^ on day 1) plus bevacizumab (7.5 mg/kg on day 1) as first-line chemotherapy and XELIRI (capecitabine 2000 mg/m^2^/day p.o., twice daily for 14 consecutive days; irinotecan 150 mg/m^2^ on day 1) as second-line chemotherapy. Cetuximab (initial dose 400 mg/m^2^, subsequent doses 250 mg/m^2^ weekly) plus irinotecan (150 mg/m^2^, on days 1, 15, and 29) were introduced in 7-week cycles as third-line chemotherapy. For the premedication of cetuximab, dexamethasone sodium phosphate (6.6 mg), dl-chlorpheniramine maleate (5 mg), and famotidine (20 mg) were administered for about 30 min, accompanied by magnesium sulfate (40 mEq). The adverse events associated with the skin were well controlled, and Common Terminology Criteria for Adverse Events (CTCAE) version 4 grades 1–2 [16] were managed by prophylaxis with oral intake of minocycline and steroidal external agents. The patient’s serum magnesium level was checked each time to prevent hypomagnesemia induced by the cetuximab treatment. At first, no oral prophylactic supplementation of oxidative magnesium was needed. At the sixth administration of cetuximab, his serum magnesium level decreased to 1.6 mg/dL (grade 1 hypomagnesemia). At the 12th administration of cetuximab, his serum magnesium level decreased to 0.9 mg/dL (grade 2 hypomagnesemia). Intravenous supplementation of 20 mEq magnesium sulfate was administered at each treatment despite the lack of clinical symptoms. The dose of cetuximab plus irinotecan was reduced to the second level due to bone marrow suppression. The serum magnesium level remained at 0.9 mg/dL, and an ECG showed grade 1 prolonged QT or QTc intervals (Table 1). His serum magnesium level remained at 0.9 mg/dL, and no hypomagnesemia symptoms were observed by the 17th administration of cetuximab. After the treatment, however, he suddenly lost consciousness and fell down to the floor when he stood up to leave the bed. He did not respond to a verbal stimulus. He was pale with cold limbs and without a radial pulse, but no symptoms related to infusion or allergic reactions were observed. Circulatory collapse following dermatological reactions and respiratory events, such as airway obstruction and edema, were not evident. Intravenous supplementation of magnesium sulfate was administered again. He awakened 2 min after the onset of temporary LOC without any other symptoms related to hypomagnesemia, such as lethargy, tremor, tetany, and seizures. His vital signs were as follows: blood pressure 128/74 mmHg and pulse 52 beats/minute. No hemorrhage or infarction was observed in a computed tomography (CT) scan. Electrocardiogram (ECG) showed a complete right bundle branch block with sinus rhythm. The grade 1 QT and QTc intervals did not significantly change before and after the onset of LOC (Table 2). Laboratory data showed hypermagnesemia (2.8 mg/dL) due to the prophylactic administration of magnesium sulfate after drip infusion of cetuximab (Table 3). Cardiac ultrasonography displayed normal left ventricular contraction without vulvar disease, visual ejection fraction over 50%, no mitral valve relapse, no atrial valve relapse, no focal asynergy, no D-shaped left ventricle, 16 mm of fair collapse in the inferior vena cava (IVC), and an 11.6 transtricuspid pressure gradient. He was admitted for observation and further examination. One day after the onset of LOC, his serum magnesium level was 1.5 mg/dL and his clinical symptoms had completely disappeared. The patient was then discharged. No significant arrhythmia was detected by Holter ECG; thus, paroxysmal arrhythmia was excluded as an associated reason for the LOC. His serum magnesium level was checked regularly, and intravenous supplementation (40 mEq magnesium sulfate) was administered twice weekly for 2 weeks and once weekly for 5 weeks (nine times total) in the outpatient unit. Afterwards, administration of cetuximab was discontinued. The serum magnesium level returned to a normal range (1.7–2.5 mg/dL) after approximately 6 weeks. Follow-up CT scans detected enlarged metastatic lymph nodes in the left supraclavicular and para-aortic region and newly emerging lumbar vertebral metastasis, showing progressive disease. Regorafenib and TAS-102 (trifluridine tipiracil hydrochloride) were introduced sequentially for 6 months. Five months after the final treatment of TAS-102, he died of his primary disease, which reflected a survival period of 4 years and 6 months since the beginning of treatment.

**Table 1:** Time course of serum magnesium level, ECG, level of dose reduction of chemotherapy, and supplementation of magnesium

Total number of administrations	Mg sulfate supplementation	Dose reduction	Abnormality on ECG	Serum Mg	CTCAE grade
12	20 mEq	None	No	0.9 mg/dL	2
13	20 mEq	1st level	No	1.0 mg/dL	2
14	20 mEq	1st level	No	0.9 mg/dL	2
15	40 mEq	2nd level	No	0.9 mg/dL	2
16	40 mEq	2nd level	No	0.9 mg/dL	2
17	40 mEq	2nd level	No	0.9 mg/dL	2

*Mg* magnesium, irinotecan, *ECG* electrocardiogram, *CTCAE* Common Terminology Criteria for Adverse Events

**Table 2:** QT and QTc intervals on electrocardiogram before and after onset of loss of consciousness (LOC)

Occasion	QT (msec)	QTc (msec)
At the colectomy (4 years before LOC)	460	435
One week before LOC	458	445
Before drip infusion	462	455
Just after LOC	502	472
After admission	500	478
The next day after LOC	458	458

**Table 3:** Laboratory data before administration of cetuximab and just after loss of consciousness

	Before administration	Just after LOC
WBC (/μL)	4410	3400
RBC (×10^4^/μL)	299	276
Hgb (g/dL)	9.6	8.6
PLT (×10^4^/μL)	16.8	15.3
TP (g/dL)	5.3	
Alb (g/dL)	3.2	
T-Bil (mg/dL)	0.33	
AST (mU/mL)	31	27
ALT (mU/mL)	29	26
LDH (mU/mL)	252	229
ALP (mU/mL)	266	250
CRP (mg/dL)	0.16	0.13
Na (mmol/L)	143	141
K (mmol/L)	4.2	3.8
Cl (mmol/L)	111	113
Ca (mg/dL)	8.0	7.2
IP (mg/dL)	2.7	2.5
Mg (mg/dL)	0.9	2.8
BUN (mg/dL)	11	10.0
Cr (mg/dL)	1.00	0.81
eGFR (mL/min/1.73 m^2^)	56.6	71.3

*WBC* white blood cell, *RBC* red blood cell, *Hgb* hemoglobin, *PLT* platelet, *TP* total protein, *Alb* albumin, *T-Bil* total bilirubin, *AST* aspartate aminotransferase, *ALT* alanine aminotransferase, *LDH* lactate dehydrogenase, *ALP* alkaline phosphatase, *CRP* C-reactive protein, *Na* sodium, *K* potassium, *Cl* chloride, *Ca* calcium, *P* phosphorus, *Mg* magnesium, *BUN* blood urea nitrogen, *Cr* creatinine, *eGFR* estimated glomerular filtration rate

## Discussion

This report presented a case of a patient who lost consciousness just after treatment with anti-EGFR antibody despite management of hypomagnesemia (0.9 mg/dL). Magnesium deficiency may cause LOC in connection with nervous or muscular disorder, but no direct evidence was observed in this case. Arrhythmia may lead to LOC by affecting the cardiovascular or nervous systems. Cardiac ultrasonography, Holter ECG, and brain CT findings showed no abnormalities, except slightly prolonged QTc intervals in the ECG before treatment. This case report serves as a reminder to clinicians that LOC can be induced without severe hypomagnesemia or QTc prolongation during anti-EGFR antibody treatment.

It is rarely reported that treatment with anti-EGFR antibody can cause LOC. Out of 4603 cases (11,069 administrations) from September 19, 2008, to October 2, 2011, only 3 cases were recorded in the reports of adverse events in safety information in Japan [14, 15]. Among these three patients, the first lost consciousness accompanied by allergic symptoms 5 min after the administration of cetuximab; therefore, this was likely an infusion reaction. The second patient showed LOC 1 day after the administration of cetuximab. The report on the third patient did not include detailed information about the period of onset of LOC (Table 4). Adverse events such as syncope and LOC have been reported as symptoms induced by infusion reaction. Most infusion-related reactions happen early in the infusion or within 1 h of the end of the infusion, during administration of the initial cetuximab treatment. Considering the onset of LOC after complete infusion of cetuximab at the 17th treatment, it is unlikely that an infusion reaction is implicated in the onset of LOC in this patient. However, several patients have shown severe infusion reactions after several cetuximab treatments [17]. No symptoms related to infusion reaction or allergic reaction were recognized outside of LOC. These reactions usually begin with circulatory collapse following dermatological reactions and respiratory events, such as airway obstruction and edema, all of which were not evident in this patient. Changes in vital signs, such as blood pressure and pulse, were also frequently observed during these reactions; however, vital signs of the patient were stable during LOC. All things considered, it is unlikely that infusion or allergic reactions were involved in the onset of LOC in this patient.

**Table 4:** Details of cases of loss of consciousness on the manufacturer’s reports of adverse events

Sex	Age	Relationship with Cet	Outcome	Period from the infusion	Diagnosis (metastatic site)	Concomitant drug	Under investigation
Female	60	Yes	Recovered	5 min	Colon cancer (peritoneum, lymph nodes)	Dexamethasone, Azasetron	
Male	80	Yes	Recovered	1 day	Colon cancer (liver)	Chlorpheniramine, Dexamethasone, Granisetron	
Male	70	Yes	Not recovered	Not mentioned	Colon cancer	Fluorouracil, calcium folinate, oxaliplatin	Yes

*Cet* cetuximab, *DEX* dexamethasone sodium phosphate, *Azasetron* azasetron hydrochloride, *Chlorpheniramine* chlorpheniramine maleate, *Granisetron* granisetron hydrochloride

Hypomagnesemia is likely induced by several drugs including anti-EGFR antibodies (Table 5) [18]. Grade 2 hypomagnesemia in this patient may have been involved in LOC despite the lack of evidence. Magnesium plays a variety of physiological and biochemical roles in the body. Symptoms of magnesium deficiency may affect nerves, muscles, mental status, and circulatory or digestive organs. In cases of nervous or muscular disorder, neurologic hypersensitivity symptoms develop, including tremor, myoclonus, muscle weakness, ataxia, tetany, muscle cramps, dizziness, nystagmus, convulsions, or coma. Among these symptoms, general fatigue is often found in hypomagnesemia and is a common clinical manifestation even in cancer patients without hypomagnesemia. Therefore, it is difficult to predict hypomagnesemia from clinical symptoms. The specific mechanism of hypomagnesemia induced by anti-EGFR antibodies is not well known. The EGFR pathway is inhibited by anti-EGFR antibodies, which leads to an inhibition of magnesium reabsorption by transient receptor potential cation channel, subfamily M, member 6 (TRPM6) in the renal tubules, accompanying hypomagnesemia [8, 19]. In addition, malabsorption of magnesium in the intestinal tract [8] and tubular impairment by the precipitation of the monoclonal antibody [20] are involved in this mechanism. The incidence of hypomagnesemia in cetuximab plus irinotecan treatment was 33.8% for any grade and 8.4% for grades 3/4 [21]. However, few cases develop grade 4 hypomagnesemia, which forces discontinuation of antibody therapy [8]. The longer patients are treated with cetuximab, the more often they experience hypomagnesemia [22]. In our patient, grade 1 hypomagnesemia occurred 1 month after the administration of cetuximab, while LOC was observed 5 months after its administration.

**Table 5:** Drugs associated with hypomagnesemia

Diuretics (furosemide, thiazide)	
Epidermal growth factor receptor inhibitors (cetuximab)	
Proton pump inhibitors (all, such as omeprazole)	
Platinum derivatives (cisplatin, carboplatin)	
Calcineurin inhibitors (cyclosporin A, tacrolimus)	
Antimicrobials (aminoglycosides, pentimidine, rapamycin, amphotericin B, foscarnet)	

Acquired long QT interval is a disorder of cardiac repolarization that is most often induced by specific drugs, hypokalemia, or hypomagnesemia that may precipitate torsade de pointes and cause syncope and sudden cardiac death [10, 11]. Normal QTc values in the adult population are 350–450 ms and 360–460 ms for males and females, respectively [23]. The patient showed a slightly prolonged QTc (> 450 ms) that was classified as a grade 1 adverse event. Syncope is the sudden transient loss of consciousness and is common both in the general population and in patients in acute care settings [12, 13]. According to the Canadian Syncope Risk Score to predict serious adverse events, in 5010 patients with syncope, a QTc interval > 480 ms was a risk factor for the development of serious adverse events. Interestingly, among 5010 patients with syncope, the mean QTc interval in 4904 patients, who did not show serious adverse events, was 432.4 ms, whereas in 106 patients, who developed serious adverse events, it was 467.4 ms, suggesting a prolonged QTc interval < 480 ms may lead to LOC. Considering these data, a prolonged QTc interval can be involved in the onset of LOC in this patient, although it was 472 ms (grade 1 adverse event) just after the onset of LOC.

The serum magnesium level was monitored from the beginning of treatment, and magnesium was supplied by drip infusion of magnesium sulfate prior to the manifestation of the clinical symptoms. The level of magnesium between serum and cells is not balanced immediately; thus, the gross levels of magnesium in the body may be insufficient despite a normal level of magnesium in the serum. In addition, it takes time for recovery after the manifestation of serum hypomagnesemia because intracellular magnesium is depleted. The safety information for cetuximab [17] recommends periodic monitoring for hypomagnesemia during and for at least 8 weeks following the completion of cetuximab therapy. Our patient took approximately 6 weeks to show a level of magnesium within the normal range after discontinuation of cetuximab. In order to prevent a decrease in serum magnesium levels, oral intake of magnesium oxide could be useful from the beginning of cetuximab treatment [24]. Intravenous supplementation with magnesium sulfate is recommended because oral supplementation of magnesium does not provide efficient absorption [8, 22]; however, there is no consensus regarding methods to adjust serum magnesium levels.

## Conclusions

We experienced a patient with LOC accompanied by mild hypomagnesemia and QTc prolongation under treatment with anti-EGFR antibody for mCRC. The LOC occurred under careful monitoring of the serum level of magnesium with supplementation and ECG. This case report reminds clinicians that LOC can be induced without severe hypomagnesemia or QTc prolongation during treatment with anti-EGFR antibodies.

## Data Availability

Not applicable.

## Abbreviations

ECG: Electrocardiogram
EGFR: Epidermal growth factor receptor
LOC: Loss of consciousness
TAS-102: Trifluridine tipiracil hydrochloride
